# Resolutions Passed by the Chicago Veterinary Society

**Published:** 1897-06

**Authors:** 


### RESOLUTIONS PASSED BY THE CHICAGO VETERINARY SOCIETY.

The following resolutions were passed by the Chicago Veterinary Society at their last regular meeting, May 13th:

Whereas, The Governor having appointed a non-graduate to the position of State Veterinarian for the State of Illinois, be it

*Resolved*, That it is the sense of this meeting that the Assistant State Veterinarians among our members do tender their resignations as such, and further, that none of our members do apply for or accept a similar position under the present incumbent; be it further

*Resolved*, That we as a Society do hereby apply to all qualified practitioners in this State to follow a similar course of action in order to uphold the integrity of the veterinary profession.

The members of the Chicago Veterinary Society positively refuse to have any business intercourse with the present appointee in any manner whatever, and are using every effort to make the Governor reconsider his appointment.

Foreign countries will soon take this matter up, owing to the incumbent being a non-graduate, and without doubt, interested parties will soon discover that the exportation of live-stock from this State will be interfered with. A number of the members of this Society have held the office of Assistant State Veterinarian for a number of years, and are men of the highest rank in our profession, most of whom have publicly expressed their intentions of sending their resignations to proper parties, and have voiced their desires that no other practitioner will so degrade the profession as to take a vacancy under the present chief.

